# Effects of Long-Term Cultivation on Medium with Alpha-Ketoglutarate Supplementation on Metabolic Processes of* Saccharomyces cerevisiae*

**DOI:** 10.1155/2017/8754879

**Published:** 2017-10-17

**Authors:** Nadia Burdyliuk, Maria Bayliak

**Affiliations:** Department of Biochemistry and Biotechnology, Vasyl Stefanyk Precarpathian National University, 57 Shevchenko Str., Ivano-Frankivsk 76018, Ukraine

## Abstract

During last years, alpha-ketoglutarate (AKG), an important intermediate in the Krebs cycle, has been intensively studied as a dietary supplement with stress-protective and potential antiaging effects. Here, we examined the effects of exogenous AKG on metabolic processes and survival of yeast* Saccharomyces cerevisiae* during long-term cultivation. Growth on AKG had no effect on the total cell number but increased the number of reproductively active cells at the late days of cultivation (from day 7 to day 15). A gradual increase in levels of total protein, glycogen, and trehalose was found over 7-day cultivation with more pronounced effects in AKG-grown cells. In control cells, metabolic activity and the activities of superoxide dismutase and catalase decreased, whereas levels of carbonyl proteins and low-molecular-mass thiols increased during 7-day cultivation. This suggests development of oxidative stress in stationary phase cells. Meanwhile, stationary phase cells cultured on AKG possessed higher levels of low-molecular-mass thiols and lower levels of carbonyl proteins and *α*-dicarbonyl compounds when compared to control ones. Collectively, higher levels of storage carbohydrates and an activation of antioxidant defense with diminishing oxidative protein damage can prevent a loss of reproductive ability in yeast cells during long-term cultivation on AKG-supplemented medium.

## 1. Introduction

Alpha-ketoglutarate (AKG), an anion of alpha-ketoglutaric acid, is an important intermediate in the Krebs cycle, which couples amino acid metabolism with glucose oxidation. Studies on animal models have shown that dietary AKG supplementation confers many favorable effects on metabolism, stress resistance, and functional decline of various age-related processes [1–3]. The beneficial effects of AKG are mainly explained by its role as a precursor of certain amino acids such as glutamate, glutamine, leucine, and proline [1, 4]. In addition, AKG can inhibit protein catabolism and activate anabolic processes in animal tissues [5]. Furthermore, exogenous AKG can be involved in the Krebs cycle and thereby intensify mitochondrial respiration [6]. As a result, an increased generation of reactive oxygen species (ROS), by-products of respiratory metabolism, may occur and that leads to oxidative stress development [7]. Thus, the higher ROS levels were shown in nematode* Caenorhabditis elegans* grown on AKG-supplemented medium [8].

Previous studies suggest that exogenous AKG can induce oxidative stress of low intensity which is accompanied by an activation of defense systems. The mentioned stimulation of protective mechanisms was supposed to be responsible for higher tolerance of AKG-treated organisms to challenge strong oxidative or other kinds of stresses. It may also contribute to preventing functional decline with age [2, 3, 9]. In particular, the AKG-supplemented food prevents an age-related increase in free radical damage to biomolecules in aged mice [3] and increases stress resistance in* Drosophila melanogaster* flies [9]. Meanwhile, AKG affected differently oxidative stress parameters and levels of storage macromolecules in young and old* D. melanogaster* [2, 9]. We reported recently that growth on AKG-supplemented medium increased resistance of exponentially growing yeast* Saccharomyces cerevisiae* to oxidative stress induced by hydrogen peroxide. Moreover, AKG-grown yeast cells showed higher activities of catalase and glutathione reductase and higher levels of thiol-containing compounds, indicating an activation of antioxidant system [10].

Since we already know that the functioning of antioxidant system is strongly connected with carbon/energetic metabolism and protein synthesis, this study aimed to examine effects of AKG on* S. cerevisiae* cells during long-term cultivation by analyzing various metabolic parameters and indicators of ROS homeostasis. Among metabolic parameters, the main focus was made on total metabolic activity and levels of protein and stored carbohydrates, glycogen, and trehalose, which play important roles in long-term yeast viability [11–14]. Prolonged cultivation leads to chronological aging of yeast cultures, which means a loss of reproductive ability that is followed by death of yeast cells [15, 16]. Based on data showing that AKG has antiaging effects in multicellular animals, we tested if AKG was able to slow down aging in* S. cerevisiae*. For this aim, the chronological lifespan of yeasts cultured with and without of AKG in the medium was determined by counting the number of reproductive active cells.

## 2. Materials and Methods

### 2.1. Reagents

Phenylmethylsulfonyl fluoride (PMSF), ethylenediamine-tetraacetic acid (EDTA), 2,4-dinitrophenylhydrazine (DNPH), 5,5′-dithiobis(2-nitrobenzoic acid) (DTNB), 2,3,5-triphenyltetrazolium chloride (TTC), Girard's reagent T, amyloglucosidase (#10115), trehalase (#T8778), peptone, and yeast extract were obtained from Sigma-Aldrich Corporation (USA); N,N,N′,N′-tetramethylethylenediamine (TEMED) and quercetin were from Reanal (Hungary); disodium salt of alpha-ketoglutaric acid (99% purity) was from Protista AB (Sweden); all reagents for determination of glucose and triacylglycerides (TAG), aspartate aminotransferase (AST), and alanine aminotransferase (ALT) activities were from PZ Cormay S.A. (Łomianki, Poland). All other reagents were obtained from local suppliers (Ukraine) and were of analytical grade.

### 2.2. Growth Conditions and Stationary Phase Survival

The* S. cerevisiae* strain YPH250 (wide type,* MAT ***a*** trp1-*Δ*1 his3-*Δ*200 lys2-801 leu2-*Δ*1 ade2-101 ura3-52*) used in this study was kindly provided by Dr. Youshiharu Inoue (Kyoto University, Japan). Cells were grown at 28°C with agitation at 175 rpm in a liquid YPD medium containing 1% (w/v) yeast extract, 2% (w/v) peptone, 2% (w/v) glucose, and 10 mM disodium salt of *α*-ketoglutaric acid (AKG). The initial cell titre in the medium was about 0.3 × 10^6^ cells/ml.

The viability of yeast cells was determined by colony-forming assay [15]. For that, aliquots (100 *μ*l) were sampled from each of yeast cultures, serially diluted, and plated in triplicate on YPD medium with agar. The viability was assessed as an ability of an individual yeast cell to reproduce and form a colony (a colony-forming unit or CFU) on YPD agar. Cells were grown at 28°C for three days before colony counting. The total number of cells in yeast cultures was counted in a Goryaev chamber after 50-fold dilution under light microscope.

### 2.3. Preparation of Cell-Free Extracts and Assay of Enzyme Activities and Protein Concentration

The total number of cells in yeast cultures was counted at 18 h (late exponential phase), at 42 h (early stationary phase), and at day 7 (late stationary phase) of growth. After counting, cells from experimental cultures were collected by centrifugation at room temperature (5 min, 3000*g*) and washed with 50 mM potassium phosphate buffer (КPi, pH 7.0). The yeast pellets were weighed and resuspended in lysis buffer (50 mM КPi, 1 mM PMSF, and 0.5 mM EDTA). Cell extracts were prepared by vigorous vortexing yeast suspensions with glass beads (0.5 mm) as described earlier [17] and kept on ice for an immediate use. The biochemical parameters were measured with spectrophotometers Spekol-211 (Carl Zeiss, Jena, Germany) or SF-46 (LOMO, Leningrad, Russia).

The measurement of activities of superoxide dismutase (SOD, EC 1.15.1.1) and catalase (EC 1.11.1.6) was conducted as described earlier [17].

Activities of aspartate aminotransferase (ASAT, EC 2.6.1.1) and alanine aminotransferase (ALAT, EC 2.6.1.2) were measured by modified methods according to International Federation of Clinical Chemistry (IFCC) using Liquick Cor-ASAT and Liquick Cor-ALAT kits, respectively.

Soluble protein concentration was determined by the Coomassie brilliant blue G-250 dye-binding method [18] with bovine serum albumin as a standard.

### 2.4. Determination of Levels of Low-Molecular-Mass Thiols

To determine the levels of low-molecular-mass thiols (L-SH), aliquots of cell extracts were mixed with trichloroacetic acid to the final acid concentration of 10% and centrifuged (16,000*g*, 5 min, 21°C) to remove pelleted protein and the final supernatants were used for assays.

Free thiol-containing compounds were measured in the obtained protein-free supernatants as absorbance of thiol conjugates with 5,5′-dithiobis-2-nitrobenzoic acid (DTNB) at 412 nm [19]. Aliquots of supernatants (75 *μ*l) were incubated with 20 *μ*M DTNB in 50 mM KPi buffer (pH 8.0) in a final volume of 1.5 ml for 30 min. In blanks, supernatant was substituted for the respective volume of trichloroacetic acid solution. Absorption was read at 412 nm and a molar extinction coefficient of 14 × 10^3^ M^−1^cm^−1^ was used to calculate the thiol level. Thiol levels were expressed as nanomoles of SH-groups per 10^8^ cells.

### 2.5. Determination of *α*-Dicarbonyl Compounds and Carbonyl Proteins

The content of carbonyl groups in proteins (CP) was measured by determining the amount of 2,4-dinitrophenylhydrazone formed upon reaction with 2,4-DNPH as described previously [17]. Carbonyl content was calculated from absorbance of 2,4-dinitrophenylhydrazone measured at 370 nm using an extinction coefficient of 22 mM^−1^cm^−1^. The results are expressed in nanomoles per mg of protein.

*α*-Dicarbonyl compounds (DC) were measured by the Girard-T reaction [20]. The absorbance of the disubstituted compound, which is formed by binding of two Girard's reagent T molecules to dicarbonyl groups, was measured at a maximum absorption wavelength of 325 nm using an extinction coefficient of 18.8 mM^−1^cm^−1^ for glyoxal [16]. The results are expressed in nanomoles of glyoxal equivalents per mg of soluble protein.

### 2.6. Determination of Carbohydrate and Triacylglyceride Levels

For experiments, 2 × 10^8^ cells from experimental cultures were harvested and washed twice with distilled water. The yeast pellets were resuspended in 50 mM Na-acetate buffer (pH 5.2) for carbohydrate measurement or in PBST buffer (10 mM Na_2_HPO_4_, 2 mM КН_2_РО_4_, 137 mМ NaCl, 2,7 mM KCl, and 0.05% Triton X-100, pH 7.4) for triacylglyceride assay. The cell suspensions were vortexed for 7 min with glass beads (2 : 1, w/w, yeasts/beads) at room temperature and heated at 70°C for 5 min to inactivate endogenous enzymes. Cell debris was removed by centrifugation for 15 min at 16000*g* at room temperature. The cell extracts were used for the further assays.

The content of free intracellular glucose was measured by glucose oxidase assay using a diagnostic kit, Liquick Cor-glucose, following the manufacturer's instructions. The content of glycogen and trehalose in cell extracts was determined by measuring glucose released by amyloglucosidase or trehalase, respectively [21]. For that, 10 *μ*l of the sample was incubated overnight with 10 *μ*l (1 U/ml) amyloglucosidase or 10 *μ*l (0.025 U/ml) trehalase with slight agitation at 37°C. The amount of glucose released from trehalose and glycogen was determined by Liquick Cor-glucose kit. The levels of trehalose and glycogen were calculated by subtraction of the amount of free intracellular glucose from the total glucose content in samples after overnight incubation with the enzymes. Standard glucose solutions in a concentration range from 2 to 20 *μ*g/ml were used to calculate glucose content in yeast cell extracts. Results are expressed as *μ*g glucose per 10^8^ cells.

Triacylglyceride (TAG) levels were measured using a diagnostic kit, Liquick Cor-TG, following the manufacturer's instructions. Standard TAG solutions in the concentration range from 3 to 30 *μ*g/ml were used for calculation of TAG content in yeast cell extracts. Results are expressed as *μ*g TAG per 10^8^ cells.

### 2.7. Determination of Metabolic Activity

To evaluate metabolic activity of the yeast, 2,3,5-triphenyltetrazolium chloride was used [16]. For experiments, 3 × 10^8^ cells from experimental cultures were harvested and washed twice with distilled water. The yeast pellets were resuspended in 1 ml of 50 mM KPi (pH 7.0) and mixed with 0.35 ml of 0.5% 2,3,5-triphenyltetrazolium chloride (TTC). Metabolically active cells are able to reduce the dye to a water-insoluble red formazan that was extracted from the cells with ethanol/acetone mixture (2 : 1), and the absorbance of this solution was measured at 485 nm [22]. The results are expressed as OD_485_ units per 10^8^ cells.

### 2.8. Statistical Analysis

Experimental data are expressed as the mean value of 4–8 independent experiments ± the standard error of the mean (SEM). Comparison between means was performed using a two-tailed Student's *t*-test or analysis of variance (ANOVA) followed by the Student-Newman-Keuls test.

## 3. Results

### 3.1. Alpha-Ketoglutarate Increases Reproductive Ability of Yeast Cells in Aged Cultures

Yeast cells were cultivated in rich YPD medium. In the YPD medium, yeast cells are actively budding and produce a large amount of young cells. Thus, the population always contains a mixture of young and old yeast cells. It causes troubles with an accurate estimation of maximal yeast lifespan. Furthermore, regrowth of yeast cells can be observed due to utilizing the nutrients released by dead cells after the culture reaches the maximum density [15]. Despite these limitations, the cultivation in YPD medium allows estimating reliably the effects of different supplements on aging rate of yeast populations [16, 23, 24]. In particular, we previously found the ability of* Rhodiola rosea* extract to increase yeast viability in the YPD medium under prolonged cultivation [24].

**Figure 1:**
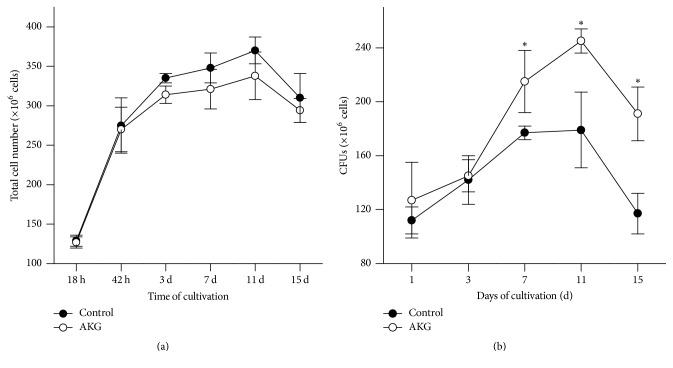
Effects of growth conditions on total cell number (a) and number of colony-forming units (CFUs) (b) in* S. cerevisiae *cultures. Yeast cells were grown during 7 days in the control medium or in the medium supplemented with 10 mM AKG. The starting cell concentration in the medium was about 0.3 × 10^6^ cells/ml. Results are shown as means ± SEM (*n* = 5–7). ^*∗*^Significantly different from respective control values with *P* < 0.05 using Student's* t*-test.

Earlier, we examined the effects of different AKG concentrations on yeast growth in the YPD medium and oxidative stress resistance of exponentially growing yeast cells [10]. We found that at the concentration of 10 mM AKG had no effect on yeast growth but it enhanced endogenous antioxidant defense and increased yeast resistance to oxidative stress. In addition, the medium supplemented with sodium chloride was used as an additional control to exclude effects of sodium ions. We did not found any effects of 20 mM NaCl on antioxidant defense system and yeast stress resistance. In this study, the total cell number and number of reproductively active cells were measured in yeast cultures grown on YPD medium supplemented with 10 mM AKG. In both control and AKG-supplemented cultures, the total cell number was changed in a similar way during cultivation (Figure 1(a)). At 18 h of growth, yeast cultures had the lowest cell number, which later increased approximately twice at 42 h (*P* < 0.005). Then, the number of cells increased slightly at day 3 (*P* < 0.05) and remained unchanged till day 11. By 16% and 13%, reductions in total cell number were observed at day 15 in the control and experimental cultures, respectively, compared with day 11 (Figure 1(a)). The number of reproductively active cells in yeast cultures was assessed by the ability of a cell to form a colony on YPD agar plates. As seen in Figure 1(b), colony-forming ability increased from day 1 to day 11 in both yeast cultures. It correlated to higher total cell number in these cultures (Figure 1(a)). However, total number of cells and number of reproductively active cells were coincided only at day 1. At further cultivation, the number of reproductive active cells was lower than the total cell number, suggesting a decrease in reproductive potential of yeast cultures with an age. Colony-forming abilities were similar in the control and AKG-supplemented cultures at days 1–3. However, AKG-supplemented cultures had by 25% and 35% more reproductively active cells at days 7 and 11 compared to the control ones. At day 15, reproductive ability of yeast cells was markedly reduced in comparison with day 11, but the control cells demonstrated more substantial loss of ability to reproduce than AKG-grown ones. Upon the similar total cell number, AKG-supplemented cultures had by 60% higher number of reproductive cells at day 15 than the control ones.

### 3.2. Cultivation on AKG Increases Cell Biomass and Protein Level in Yeast Cells

**Figure 2:**
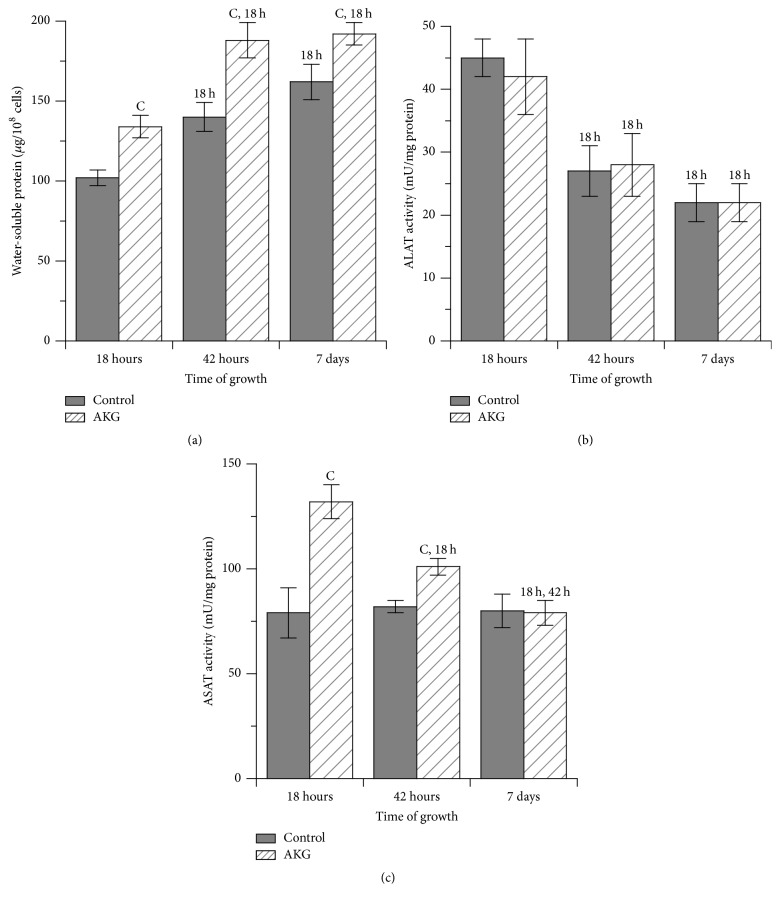
Levels of water-soluble protein (a), ALAT activity (b), and ASAT activity (c) in yeast* S. cerevisiae* cells grown during 7 days in the control medium or in the medium supplemented with 10 mM AKG. Results are shown as means ± SEM (*n* = 5–8). C, significantly different from respective control values with *P* < 0.01 using Student's* t*-test; 18 h, significantly different from respective values at 18 h, and 42 h, different from respective values at 42 h with *P* < 0.05 using Student-Newman-Keuls test.

Protective effects of AKG on yeast viability were observed already at day 7 of cultivation (Figure 1(b)). Therefore, we examined several biochemical parameters characterizing the intensity of metabolic processes in* S. cerevisiae* cells cultivated with and without 10 mM AKG during a 7-day period. The parameters were measured in cells collected at late exponential phase (18 h), early stationary phase (42 h), and late stationary phase of growth (7 d). In Figure 2, we show level of water-soluble protein and activities of two enzymes of amino acid metabolism, ALAT and ASAT. Level of water-soluble protein increased over a time of cultivation on both control and AKG-supplemented media (Figure 2(a)). As a result, control and AKG-cultivated cells had 60% and 43% higher protein levels, respectively, at day 7 compared with 18 h of growth. Meanwhile, the cultivation on AKG-supplemented medium promoted higher protein levels in yeast cells. In particular, AKG-grown cells displayed 31% and 19% higher protein levels at 18 h and day 7, respectively, compared to the control ones at the same time points. Notably, in parallel with an increased protein level at exponential phase, AKG-grown cells had 15% higher wet biomass than the control ones (Table 1).

The activities of ALAT were similar in the control and AKG-grown cells over the cultivation period (Figure 2(b)). Yeast cells demonstrated the highest ALAT activity at 18 h of growth with further decreasing enzyme activity by ~40% and 50% at 42 h and day 7, respectively. The activity of ASAT showed different activity patterns in the control and AKG-cultivated cells (Figure 2(c)). At 18 h of growth, AKG-grown cells had 40% higher ASAT activity as compared to the control cells. The ASAT activity was unchanged in the control cells during next days of cultivation, whereas in AKG-grown cells, the activity demonstrated a time-dependent decrease, being 23% and 40% lower at 42 h and day 7, respectively, compared with the one at 18 h.

### 3.3. AKG Increases Levels of Storage Carbohydrates in Yeast Cells

**Figure 3:**
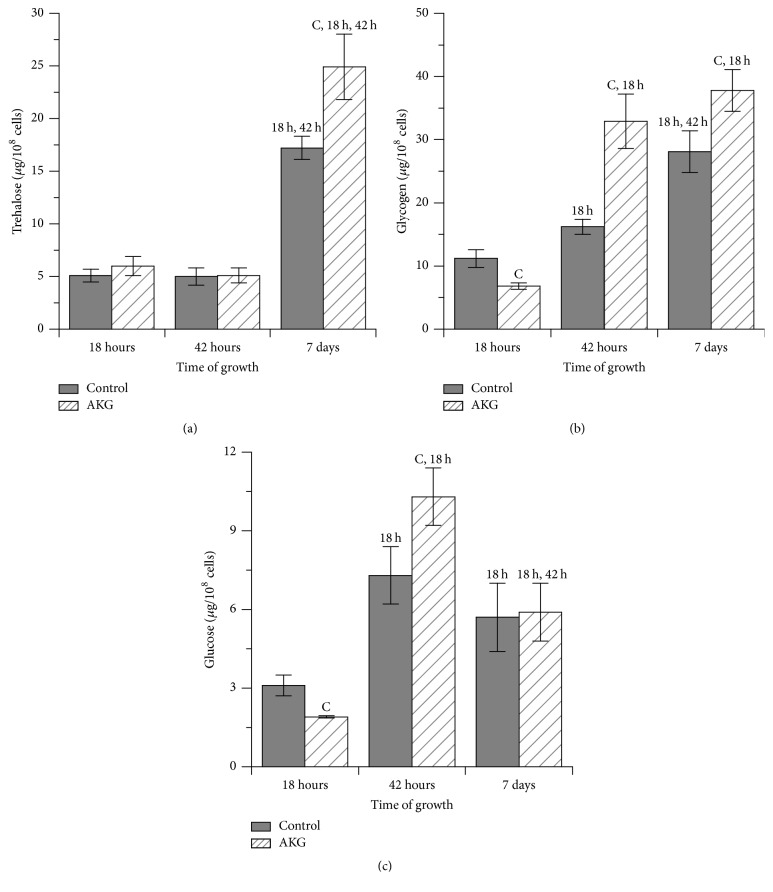
Levels of reserve carbohydrates, trehalose (a) and glycogen (b), and free glucose (c) in* S. cerevisiae* cells grown during 7 days in the control medium or in the medium supplemented with 10 mM AKG. Results are shown as means ± SEM (*n* = 6–8). Statistic as in Figure 2.

In the control and AKG-supplemented cultures, content of storage carbohydrates, trehalose and glycogen, rose over experimental time (Figures 3(a) and 3(b)). Trehalose levels were similar at 18 h and 42 h of cultivation in both yeast cultures (Figure 3(a)). At day 7, 3.3-fold and 4.6-fold increases in trehalose level were observed in cells cultivated with and without AKG, respectively, when compared with the same yeast cultures at 18 h of cultivation. When comparing control and AKG-grown yeast cultures within day 7, in the supplemented yeasts, trehalose level was 1.4-fold higher. The level of glycogen was 40% lower in exponential phase cells grown with AKG supplementation compared to the control ones (Figure 3(b)). During next days of cultivation, glycogen seemed to be significantly accumulated in yeast cells from both cultures, but control cells showed less glycogen than AKG-supplemented ones. Thus, glycogen levels were 1.4-fold and 2.5-fold higher in the control cells and 4.8-fold and 5.5-fold higher in AKG-cultivated cells at 42 h and day 7, respectively, as compared to values at 18 h of growth. A bell-shaped mode of glucose content was observed for yeast cells (Figure 3(c)). In both control and AKG-grown cells, glucose level was the lowest at 18 h and demonstrated a significant increase at 42 h (2.4-fold in the control and 5.5-fold in AKG-grown cells). Then, at day 7, in AKG-supplemented yeasts, glucose content became lower than that observed previously at 42 h of cultivation, but it was still higher than at 18 h, whereas for the control yeasts, the difference was not significant. It should be noted that AKG-grown cells displayed 39% lower and 40% higher free glucose level at 18 h and 42 h, respectively, than control cells at the same cultivation time. At day 7, there was no difference in glucose levels in cells from both cultures. The levels of TAG in yeast cells did not change regardless of cultivation period or AKG supplementation (Table 1).

### 3.4. Effect of AKG on Metabolic Activity and Parameters of ROS Homeostasis in Yeast Cells

**Figure 4:**
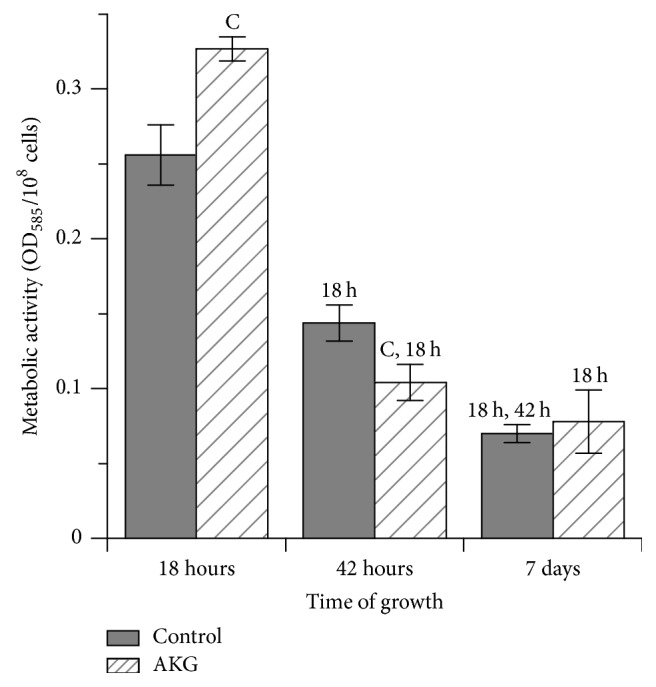
Metabolic activity of* S. cerevisiae* cells grown during 7 days in the control medium or in the medium supplemented with 10 mM AKG. Results are shown as means ± SEM (*n* = 5-6). Statistic as in Figure 2.

The total metabolic activity of yeast cells decreased over time of cultivation in both the control and AKG-supplemented media (Figure 4). In cells cultivated with AKG supplementation, metabolic activity was 1.3-fold higher at exponential phase (18 h) and 28% lower when yeasts entered stationary phase (42 h) as compared to the control cells. At day 7 of cultivation, cells from both control and experimental cultures had similar levels of this parameter.

**Figure 5:**
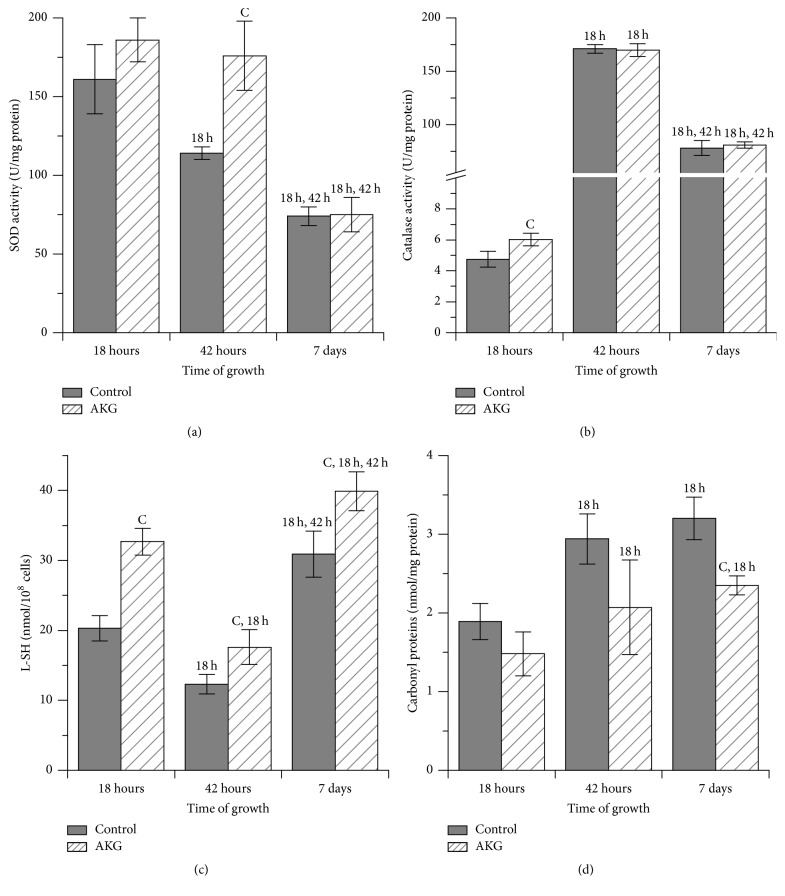
Activities of SOD (a) and catalase (b) and levels of low-molecular-mass thiols (c) and carbonyl proteins (d) in* S. cerevisiae* cells grown during 7 days in control medium or medium supplemented with 10 mM AKG. Results are shown as means ± SEM (*n* = 5–7). Statistic as in Figure 2.

To investigate AKG influence on the antioxidant system of yeast cells, the activities of antioxidant enzymes and oxidative stress markers were estimated. Among known antioxidant enzymes, we tested superoxide dismutase (SOD), which catalyzes dismutation of superoxide anion radical (O_2_^•−^) to hydrogen peroxide (H_2_O_2_), and catalase, which decomposes H_2_O_2_ to nontoxic water and oxygen [7]. The activities of SOD were similar in the control and AKG-grown cells at 18 h and at day 7 of cultivation (Figure 5(a)); only at 42 h, cells cultivated on AKG showed 55% higher SOD activity in comparison to the control ones. In the control yeast culture, SOD activity was the highest at 18 h and then decreased gradually with increasing age of yeast cultures. In AKG-grown cells, SOD activity reached maximal values at 42 h, where it was 2-fold higher than at 18 h, and at day 7 it decreased to the value close to that observed at 18 h. Regardless of medium type, catalase activity was the lowest at 18 h and the highest at 42 h of cultivation with a moderate decrease (~2-fold) at day 7 (Figure 5(b)). Significant difference in catalase activity between control and experimental cells was observed only at 18 h, where AKG-grown cells had 26% higher catalase activity than the control ones. The levels of oxidative stress markers, low-molecular-mass thiols (L-SH) (Figure 5(c)) and carbonyl proteins (CP) (Figure 5(d)), rose over a cultivation time in cells grown in medium with or without 10 mM AKG. At the same time, AKG-grown cells had 61%, 43%, and 29% higher L-SH levels at 18 h, 42 h, and day 7, respectively, as compared to the control cells at the same time points. The level of CP did not differ in cells from both cultures at 18 h (Figure 5(d)), but a significant increase in this parameter was observed in the control cells at next days of cultivation. Thus, the control cells had 63% and 74% higher CP level at 42 h and day 7, respectively, when compared with respective values at 18 h. At the same time, CP level in AKG-grown cells was only 31% and 37% higher at 42 h and day 7, respectively, as compared with 18 h. We also measured levels of *α*-dicarbonyl compounds (DC) in cells from 7-day cultures. These compounds together with ROS are intermediary products of nonenzymatic glycation/autoxidation of monosaccharides [16, 23]. Level of DC was 24% lower in cells grown in the presence of AKG compared with the control cells at day 7 of cultivation of yeast cultures (Table 1).

## 4. Discussion

Yeast* S. cerevisiae* is widely used to understand molecular mechanisms regulating metabolic processes in mammalian cells. It has been suggested that yeast culture at stationary phase is a good model to study aging of somatic cells in higher eukaryotic organisms, because at this stage yeasts become postmitotic cells and rely on mitochondrial respiration to maintain viability. In the yeast, process of entry into stationary phase is the best studied in glucose-containing liquid medium at 30°C and exhibits several distinct phases, including exponential phase, during which growth rates are maximal and cells use mainly glucose as carbon and energy source, and stationary phase, when all carbon energy sources are exhausted and cell number remains at relatively constant value [13, 15]. A variety of experimental data supports a key role of antioxidant defense and stored carbohydrates in the extension of stationary phase yeast lifespan [11–16, 23–27].

Alpha-ketoglutarate (AKG) is an important intermediate of many metabolic processes combining catabolic and anabolic functions. Our previous results show that the supplementation with AKG did not affect growth of* S. cerevisiae* YPH250 strain (wild type) in glucose-containing medium starting from inoculation to 72 h of cultivation [10]. Here, we demonstrated that AKG supplementation had no effect on total cell number in YPH250 cultures during a 15-day cultivation period (Figure 1(a)). Meanwhile, total cell number was changed during yeast growth in accordance with features of typical growth pattern of* S. cerevisiae* in batch culture. The cell number was the lowest at 18 h and increased about twice at 42 h. Hence, 18 h of growth corresponded to a late exponential phase of growth curve. A true stationary phase was started at day 3, because no increase in cell number was observed anymore, and this phase lasted to day 11 (Figure 1(a)). At day 15, the total cell number phase decreased, indicating a phase of culture death. As the total cell number, the number of reproductively active cells in yeast culture also depended on time of cultivation (Figure 1(b)). At exponential phase of growth (day 1), almost all cells were able to proliferate and form colonies on YPD agar plates. The positive effects of AKG on proliferative ability were observed at days 7–15, when the control cells formed lower numbers of GFUs than AKG-grown ones. Thus, AKG increased the reproductive potential of aged yeast population. Mechanisms underlying protective AKG effects could be related with modulation of metabolic pathways, where this keto acid might be involved. Our results showed that AKG promoted different metabolic changes in exponential phase and stationary phase cells.

We show here that exponentially growing yeast cells had higher values of total metabolic activity (Figure 4), protein level (Figure 2(a)), and ASAT activity (Figure 2(b)) and lower levels of glycogen (Figure 3(b)) and free glucose (Figure 3(c)) in the AKG-supplemented medium than in the control one. These results let us suggest that exogenous AKG modifies protein/amino acid and glucose metabolism in exponential phase cells. The involvement of AKG in amino acid metabolism is well established; in particular AKG was found to serve as a precursor for the biosynthesis of such amino acids as glutamate, glutamine, leucine, and proline [1, 4, 9]. Obviously, an AKG-induced increase in amino acid levels could stimulate protein synthesis, leading to the higher levels of total protein seen in yeast cells (Figure 2(a)). In support of this finding, the ability of AKG to enhance protein synthesis due to increased production of amino acids was demonstrated in intestinal porcine epithelial cells [5]. The increased protein synthesis in AKG-grown cells can be a cause of higher biomass of these cells compared to the control ones (Table 1). In addition, AKG-supplemented cells had higher protein levels than control ones at stationary phase of growth. That may indicate that AKG prevented age-associated decrease in protein synthesis and/or increase in catabolism of proteins [11]. Moreover, some studies indicate that higher concentration of amino acids in the media can promote yeast growth and lifespan extension [28, 29]. Therefore, AKG-promoted synthesis of amino acids can be important for better yeast viability in our experiments.

Apart from involvement in protein/amino acid metabolism, AKG is a Krebs cycle intermediate. Previous studies show that the externally added AKG is actively utilized via the Krebs cycle, leading to increasing mitochondrial respiration and oxidative phosphorylation [6] and ATP production [30], as well as decreasing free glucose and TAG level [2, 4, 31]. These facts can explain lower glucose and glycogen levels in AKG-grown cells, since a more active Krebs cycle (due to increase of number of intermediates) requires more molecules of acetyl-CoA formed during aerobic glucose oxidation. However, it should be noted that the Krebs cycle and respiratory chain are poorly functioning in* S. cerevisiae* cells growing exponentially at high (2%) glucose levels. Glucose is preferentially metabolized via anaerobic glycolysis to form nonfermentable carbon compounds, particularly ethanol, and it exerts a strong catabolite repression with either enzymes required for respiration or enzymes of the Krebs cycle [11, 32]. After glucose is depleted, yeast cells reorganize their metabolism from fermentation to respiration in order to utilize other carbon sources presented in the medium such as ethanol. In our previous study, we showed that the glucose concentration at 18 h of growth in the media collected from control and AKG-grown* S. cerevisiae* cultures was lower than 0.2%. It means that cells were not under glucose repression and were able to obtain energy aerobically [10]. Our present data on the total metabolic activity (Figure 4) support an increase in metabolic rate in exponential phase yeast cells grown on AKG. Since method used for determination of metabolic activity reflects mainly functioning of mitochondria [22], it may be surmised that cells grown with addition of AKG to medium could respire more actively than their counterparts grown without AKG supplementation.

Since AKG can intensify mitochondrial respiration, it may lead to increased ROS production by mitochondria. In particular, the enhanced ROS level was found in* C. elegans* grown on AKG [8]. In the response to increased ROS levels, the antioxidant system can be activated [7]. In our previous study, we showed an activation of antioxidant enzymes, catalase and glutathione reductase, together with an enhanced level of low-molecular-mass thiols, which are represented mainly by glutathione [33], in AKG-grown yeast cells at exponential phase [10]. Here, the results on exponentially growing cells (Figure 5) confirmed our previous observations.

In stationary phase, both control and AKG-grown cells were characterized by higher levels and glucose, glycogen, and trehalose (Figure 3). It was quite expected, because many studies demonstrated increased gluconeogenesis and accumulation of reserve carbohydrates in stationary phase yeast cells [11, 14, 23]. Reserve carbohydrates not only serve as energy and carbon sources in starving cells but also have stress-protective functions, especially trehalose [11–14]. The supplementation of culture medium with AKG promoted higher values of reserve carbohydrates and glucose in stationary phase cells (Figure 3). This suggests that AKG may stimulate gluconeogenesis in yeast cells. This effect can be implemented via using of AKG for synthesis of glycogenic amino acids such as glutamine [1, 4] or via metabolizing AKG in Krebs cycle to oxaloacetate, a well-known precursor in glucose biogenesis. The increased activity of ASAT in early stationary phase cells grown on AKG (Figure 2(c)) can also contribute to oxaloacetate supply for gluconeogenesis as it was shown in starved animals [8]. In turn, the increased glucose level can be directed for synthesis of reserve carbohydrates.

Loss of yeast viability during stationary phase is accompanied by reduced total metabolic activity and accumulation of oxidatively damaged biomolecules [11, 13, 16, 25, 26]. At the same time, activation of antioxidant defense and accumulation of reserve carbohydrates are considered as beneficial events for yeast cells which allow them to remain viable in aged cultures during prolonged periods of nutrient depletion [11, 13, 14, 26, 27]. Our results are in a good agreement with literature data. In the control cells, decreased SOD activity and L-SH level and increased catalase activity and level of carbonyl proteins were observed at early stationary phase (42 h of growth), compared to 18 h of growth. These changes suggest the development of oxidative stress in yeast cells entering stationary phase, which is consistent with data of other authors [16, 25, 27]. In addition, cells in stationary phase decrease their metabolic activity to maintain viability during prolonged periods [11, 13, 23]. Our results are in line with this mechanism and confirmed a decrease in total metabolic activity in cells at stationary phase compared to yeasts at exponential phase (Figure 4). At the same time, it was found earlier that slowing metabolic activity is not always accompanied with decrease in ROS production; the ROS level may be even elevated [34], and stationary phase cells suffer from chronic oxidative stress [25]. As a result, oxidative modification of biomolecules may exceed the capacity of antioxidant system. In our case, an increase in level of oxidized proteins was observed in the control cells in both early and late stationary phase, despite higher values of catalase activity and L-SH-level in these cells in comparison with exponential phase cells. Moreover, the decreased SOD activity at 42 h and at day 7 comparatively with values at 18 h and a decrease in catalase activity at day 7 comparatively to 42 h could be caused by oxidative modification of these enzymes as it was reported earlier [17, 35].

In the case of AKG-supplemented cultures, the higher levels of L-SH and less oxidative damage to proteins were observed in stationary phase yeast cells compared to the control ones (Figures 5(c) and 5(d)). The activities of SOD and catalase in AKG-grown yeasts were similar to those in the control yeasts at day 7, but at 42 h SOD activity in AKG-grown yeasts was higher than that in control ones (Figure 5(a)). In addition, AKG-grown cells had lower level of *α*-dicarbonyl compounds (DC) than control ones at day 7 (Table 1). The *α*-dicarbonyl compounds together with ROS are intermediate products of nonenzymatic glycation/autoxidation of glucose, which participate in aging processes in yeasts [16]. Higher levels of glycation products are formed at high glucose levels in the medium (2% glucose), where glucose is mainly utilized via fermentation, and it shortens chronological yeast lifespan [16, 36]. However, caloric restriction or switch of glucose metabolism from fermentation to respiration may decrease glycation and extend yeast lifespan [36]. Since above we discussed that AKG may facilitate respiration metabolism in yeast cells when glucose is available at high concentrations, this may contribute to lower DC level in aged yeast cells. In addition, lower levels of oxidized proteins (CP) and DC in AKG-supplemented cells at late days of cultivation might be a result of increasing antioxidant potential of these cells at early cultivation period, compared with the control cells. In agreement with our data, an increased superoxide generation was found to act as a signal in young* C. elegans* animals to trigger changes of gene expression which prevent or attenuate the effects of subsequent aging [37]. Thus, we can assume that AKG-induced mild oxidative stress in exponentially growing cells may result in the adaptive induction of defense mechanisms. This adaptive response can provide sufficient protection against any subsequent strong oxidative or other stresses and prevent an age-related increase of free radical damage to biomolecules, thereby contributing to better yeast viability during prolonged cultivation.

## 5. Conclusions

Our results indicate that cultivation on AKG-supplemented medium modifies metabolic processes in yeast* S. cerevisiae* and prevents a loss of reproductive potential in aged cultures. The main metabolic effects of AKG included an increase in levels of total protein and reserve carbohydrates, glycogen and trehalose, as well as an enhancement of antioxidant defense with subsequent diminishing oxidative damage to proteins in yeast cells. These changes were supposed to contribute to better survival of AKG-supplemented cells under long-term cultivation in nutrient-deprived medium. Thus, our results extend knowledge on antiaging effects of AKG found previously in animals and suggest that AKG supplementation may also be beneficial at cellular level via increasing oxidative stress resistance and maintaining proliferative potential of cells.

## Figures and Tables

**Table 1:** Wet biomass and levels of *α*-dicarbonyl compounds (DC) and triacylglycerides (TAG) in *S. cerevisiae* cells during prolonged cultivation in the control or 10 mM AKG-supplemented medium.

Parameter	Growth conditions					
	Control			10 mM AKG		
	18 h	42 h	7 d	18 h	42 h	7 d
Wet biomass (mg/10^8^ cells)	6.90 ± 0.81	NS	NS	7.90 ± 0.27^*∗*^	NS	NS
TAG, *μ*g/10^8^ cells	16.5 ± 1.1	15.2 ± 2.3	15.2 ± 1.6	15.6 ± 1.7	18.5 ± 3.1	16.7 ± 2.5
DC, nmol glyoxal equivalents/mg protein	NS	NS	7.01 ± 0.33	NS	NS	5.53 ± 0.38^*∗*^

Results are shown as means ± SEM (*n* = 6–8). ^*∗*^Significantly different from respective control values with *P* < 0.01 using Student's *t*-test. NS: not studied.
